# Specialized intensive inpatient rehabilitation is crucial and time-sensitive for functional recovery from disorders of consciousness

**DOI:** 10.3389/fneur.2023.1126532

**Published:** 2023-04-06

**Authors:** Bei Zhang, Katherine O'Brien, Jean Woo, Bradley Chi, Colton Reeh, Sheng Li, Sunil Kothari

**Affiliations:** ^1^TIRR Disorders of Consciousness Program, TIRR Memorial Hermann Hospital, Houston, TX, United States; ^2^Division of Physical Medicine and Rehabilitation, Department of Neurology, Texas Tech University Health Sciences Center, Lubbock, TX, United States; ^3^Department of Physical Medicine and Rehabilitation, McGovern Medical School, University of Texas Health Science Center at Houston, Houston, TX, United States; ^4^H. Ben Taub Department of Physical Medicine and Rehabilitation, Baylor College of Medicine, Houston, TX, United States

**Keywords:** disorders of consciousness (DOC), inpatient rehabilitation, severe brain injuries, emergence, decannulation, oral diet, effectiveness and efficiency, outcome measures

## Abstract

**Background:**

Disorders of consciousness (DoCs) after severe brain injury are considered to be conditions with dire prognosis. Despite the accumulating evidence, inpatient rehabilitation is often denied by payers referring to the Medicare/Medicaid criteria, under the assumption that such patients will not “*actively*” participate in therapy or make “*measurable improvements*.”

**Objective:**

This study aimed to report on the effectiveness and efficiency of a specialized inpatient DoC rehabilitation program based on measurable clinical parameters.

**Methods:**

A retrospective cohort study was conducted. The cohort comprised 137 patients with DoC admitted to a specialized acute inpatient rehabilitation program between January 2014 and October 2018. Patients were categorized as having been admitted at the acute stage (<=28 days post-injury), subacute stage (29–365 days following a traumatic brain injury (TBI) or 29–90 days following a non-TBI), or chronic stage (>365 days following a TBI or >90 days following a non-TBI). Outcomes included changes in level of consciousness (based on the Coma Recovery Scale–Revised (CRS-R), while also acknowledging scenarios beyond those captured by the CRS-R *via* Individualized Qualitative Behavioral Assessment and team consensus); Functional Independence Measure (FIM) levels; achievements in decannulation and initiation of oral diet; and time to those achievements.

**Results:**

The rates of emergence from a minimally conscious state were 90, 62, and 18% among patients admitted at the acute, subacute, and chronic stages, respectively. Among patients who emerged, 100, 85, and 67%, respectively, had measurable FIM scores. Approximately 60 and 20% of patients at the acute and subacute stages, respectively, required moderate assistance or less in transfer/communication/eating/grooming/upper body dressing by the time of discharge from Phase I admission. The decannulation rates were 94, 67, and 17%. The oral diet initiation rates were 70, 23, and 6%. The time to reach these achievements lengthened as chronicity increased. There was a weak positive correlation (*r*_*s*_ = 0.308) in the case of decannulation and a strong positive correlation (*r*_*s*_ = 0.606, both *p* < 0.01) in the case of oral diet between days since injury on admission and days to the achievement after admission. Patients with TBI and hypoxic brain injury had comparable recovery rates when admitted at the acute and subacute stages.

**Conclusion:**

Specialized intensive inpatient rehabilitation is crucial and time-sensitive for functional recovery from DoC caused by TBI and hypoxic–ischemic brain injury. Specific goals and different outcome measures need to be developed to appraise the benefits of acute inpatient rehabilitation for DoC.

## Introduction

Disorders of consciousness (DoCs) after severe traumatic or non-traumatic brain injury (TBI or non-TBI) are commonly considered to be conditions with dire prognosis. The spectrum of DoCs includes coma, unresponsive wakefulness syndrome/vegetative state (UWS/VS), and minimally conscious state (MCS) (1). Recently, covert consciousness (a condition also recognized as “cognitive motor dissociation” or “functional locked-in syndrome”) has been identified using advanced neuroimaging or electrophysiologic technologies in behaviorally unresponsive patients, which adds another dimension to the disease spectrum (2–4). Numerous studies worldwide have consistently shown that a continuous recovery process occurs in persons with DoC, even over a 10-year time span (5–11). The long-term outcomes in some of these patients have been surprisingly more favorable than presumed, especially among those with a traumatic etiology. A considerable proportion of those patients were able to achieve independence in at least one basic cognitive function (e.g., language/communication) and/or domain of activities of daily living (e.g., transfer, eating, dressing) over the course of 10 years post-injury (6, 7).

The road leading to recovery meanders, which is partly related to the severity of the brain injury and our limited understanding of the brain, but also arises from factors relating to healthcare access and nihilistic beliefs regarding treatments. Ten years ago, Katz et al. (11) provided evidence to support the recommendation of active and higher-intensity rehabilitation for patients with severely impaired consciousness after brain injury (11). Despite the accumulation of evidence over the years (6–11), such benefits are not commonly supported by insurance payers. The argument is that these patients do not meet the criteria of being able to “*actively* participate in 3 h of therapy per day at least 5 days per week” and are unable to make “*measurable* improvements”; therefore, they will not benefit from such a level of service (12). The prejudice regarding futility of treatments for DoCs in the minds of healthcare professionals, insurance payers, and the general population prevents these patients, who cannot advocate for themselves, from receiving opportunities for meaningful recovery, especially at an early stage after brain injury. Another contributing factor is that current regulatory measurement scales fail to capture patients' functional improvements as a result of inpatient rehabilitation services. Consequently, many patients may be misdiagnosed as having DoC or suboptimally treated due to lack of access to proper assessments and management (13, 14). Our preliminary analysis identified financial factors as the main barrier to accepting a DoC referral, and also identified a high rate of misdiagnosis in those referrals who were admitted (13). In the 2018 AAN/ACRM/NIDILRR DoC guidelines, the importance of referring a patient with DoC who is medically stable to a specialized inpatient rehabilitation program was emphasized as the top recommendation (Level B; “should be done”) (15). Overall, implementation of these guidelines remains limited. With more standardized assessment paradigms, current inpatient rehabilitative interventions have seldom been described in detail in the literature. Recent guidelines have also provided care standards and minimum competencies for rehabilitation programs providing care for persons with DoC (16). In addition, there are a limited number of such programs accepting these patients nationwide. The barriers are multifactorial and intertwined.

While the field has seen major advancements in the detection of consciousness and in standardization of assessments (2, 15), we hope to contribute by providing guidance for effective clinical rehabilitation and advocating for increased rehabilitative access for these patients. Recently, we summarized and proposed clinical approaches in the assessment of reversible causes, confounders, and mimics of DoC (17), spasticity management (18), and the application of GABAergic medication trials (19). It is notable that meaningful improvements can be observed out of the scope of commonly used scales, such as achievement of decannulation and initiation of oral diet, thereby facilitating remaining voluntary motor control, etc. A primary focus of the present study was to report on the effectiveness of specialized intensive rehabilitative services for DoC related to TBI and non-TBI at various stages post-injury based on measurable clinical parameters. Furthermore, as indicated in rehabilitation for stroke and other types of non-progressive brain injuries, time is a sensitive matter for neurorecovery, since the greatest pace of recovery is usually expected in the first 3–6 months post-injury. Therefore, the current study also aimed to report on the efficiency of specialized intensive rehabilitative services for functional recovery in DoC.

## Methods

This was a single-institution retrospective study. The cohort consisted of 137 patients; it was was derived from an established cohort of 146 patients, which included all patients with DoC admitted to a specialized DoC rehabilitation program from January 2014 to October 2018. Nine patients who were found to have emerged from DoC on initial evaluation upon admission were excluded from the cohort, as the study was intended to evaluate the outcomes of the DoC rehabilitation program, including improvements in level of consciousness.

### Operation of the DoC rehabilitation program

The admission criteria and screening process have previously been described in detail (17). In brief, pre-admission screening was performed to determine the appropriateness of admission to the specialized DoC program (i.e., to triage potential misdiagnosis of DoC). The program accepts all patients with DoC who are either in a USW/VS or in a MCS with or without ventilation support. Beyond this criterion, a patient needs to be medically stable for the transfer to take place.

Each patient's level of consciousness was assessed on admission and periodically (every 3–7 days) until discharge using standardized protocols, i.e., the Coma Recovery Scale–Revised (CRS-R) and the Individualized Qualitative Behavioral Assessment. It should be noted, however, that emergence from MCS (eMCS) was determined not solely by performance on these tests but also by close clinical observation and evaluation during daily encounters by the entire team and families, as some of the behavioral evidence of consciousness occurred outside of the testing scheme or was not assessed by the standardized tests [several case scenarios are reported in Zhang et al. (19)]. The assessments were performed by a dedicated group of experienced professionals. The management philosophy included addressing reversible causes of DoC (17); identifying confounders and mimics (17); managing neurological complications and general medical conditions (17, 18); improving arousal and awareness (e.g., sleep optimization, environmental enrichment, verticalization with sitting and standing schedules, mobilization, minimization of sedating or cognitive-impairing medications, use of neurostimulants, and sensory stimulation including tactile, music, and median nerve stimulation); and trialing GABAergic medications (e.g., zolpidem and/or lorazepam) for potential paradoxical stimulating responses (19). General medical management was undertaken with a systemic approach, including (but not limited to) domains such as the cardiovascular (e.g., storming), pulmonary (e.g., airway access and secretion management, ventilation/oxygenation, infection prevention), gastrointestinal (e.g., nutritional access and optimization, elimination), genitourinary (e.g., voiding, infection prevention), integumentary (e.g., skin breakdown), neuromuscular (e.g., spasticity, contracture prevention), and pain. All patients participated in at least 3 h of therapy daily, including physical, occupational, and speech therapy (provided by PT/OT/SLP), 5 days per week, with goals of identification of signs of consciousness, facilitation of the emergence of consciousness, and cardiopulmonary and neuromuscular conditioning. PT/OT provided modalities for maintenance of body mobility and joint range of motion, and helped to identify potential voluntary movements which a patient could use to answer yes/no questions (e.g., sometimes these were only trace movements of the fingers or head/neck). Physiatrists assisted PT/OT in spasticity management using injections, intrathecal baclofen, or spasmolytic medications. Respiratory therapists collaborated with SLP to work toward decannulation. SLP collaborated with OT to work on oropharyngeal exercises and oral diet initiation. Neuropsychologists communicated with the entire team and families to collate observed evidence, assessed contingent motoric and affective behaviors, collaborated with PT/OT/SLP to incorporate salient behaviors into assessment paradigms and treatments, collaborated with physiatrists on the use of neurostimulants and psychoactive agents, and provided further feedback to the team to consolidate all information and promote rehabilitative efficacy. Once a patient was noted to have emerged, the next important focus was to establish a communication system, minimize pain/discomfort, and improve quality of life. There was ongoing daily communication with nursing/caregivers and weekly family meetings were convened for updates, education, counseling, and care planning. Specialists were consulted when needed, e.g., neurosurgery for hydrocephalus and ENT for difficulty in decannulation.

A patient's first admission to the DoC program was defined as Phase I rehabilitation admission. Subsequent planned admissions were defined as Phase II, and so on. Subsequent admissions to a general brain injury rehabilitation service may occur if the patient has emerged and their level of functioning makes this appropriate. Unplanned transfer/return for medical emergencies did not constitute a new phase of admission in the study.

### Data retrieval and analysis

Basic demographic information, admission status, instances of acute unplanned transfer, and other functional information were obtained from electronic medical records (EMRs). The case mix index (CMI) is presented here as a reflection of overall medical complexity, although no designated diagnosis of DoC is involved in its calculation.

Acuity and chronicity were defined as suggested by the AAN/ACRM/NIDILRR DoC guidelines (15). “Acute stage” referred to cases <=28 days following a TBI or a non-TBI; “subacute stage” referred to cases 29–365 days following a TBI or 29–90 days following a non-TBI; and “chronic stage” referred to >365 days following a TBI or >90 days following a non-TBI. In subsequent analyses, all patients were categorized according to these three stages.

Measurable clinical outcomes included improvements in diagnostic category in terms of level of consciousness, Functional Independence Measure (FIM) scores, achievement of decannulation, and oral diet initiation. Level of consciousness was collected on admission and at final discharge (at the end of the last discharge if there were multiple phases of rehabilitation admission). The date the order was placed for decannulation (which was executed on the same day) was considered to represent the timing of achievement of decannulation. The date the order was placed for a dysphagia diet was considered to represent the timing of achievement of oral diet initiation, even if a patient might still require modifications or supplementary tube feeding. The number of patients who advanced to a regular diet was also collected. The time taken to achieve these functional goals was obtained by calculating the differences between the exact dates. FIM scores were obtained by the end of Phase I inpatient DoC rehabilitation. Measurable FIM indicated that a patient scored above 1 on any one of the items. FIM subtotal was the sum of scores on self-care, transfer, locomotion, communication, and social cognition (no sphincter control data was available), with a lowest possible score of 12 and a highest possible score of 84. The self-care domain contained five items (eating, grooming, bathing, upper body dressing, and lower body dressing), with a lowest possible score of 5 and a highest possible score of 35. The bed/chair transfer domain consisted of one item with a lowest possible score of 1 and a highest possible score of 7. The locomotion domain consisted of one item measuring walking or mobility using a wheelchair, whichever was ranked higher, with a lowest possible score of 1 and a highest possible score of 7. The communication domain contained two items (compression and expression) with a lowest possible score of 2 and a highest possible score of 14. Finally, the social cognition domain contained three items (social interaction, problem-solving, and memory) with a lowest possible score of 3 and a highest possible score of 21. The percentages of patients who required moderate assistance or less (scores ≥ 3) in bed-to-chair transfer, communication, and self-care are presented as meaningful outcomes, indicative of a meaningful reduction in care burden.

Data were analyzed in Microsoft 365 Excel and SPSS 20.0. Numerical variables are presented in the form mean±SD. In cases where the data did not follow a normal distribution, the median and interquartile are provided. Categorical variables are presented as numbers or percentages. Only data for patients admitted in the acute and subacute stages were included in the correlation analysis, as the recovery trajectory varied widely in the chronic stage. The correlations between time since injury on admission and time to achieve certain functional outcomes since admission were examined using Spearman's rank correlation. Statistical significance was set at *p* < 0.05.

## Results

The demographics and status of the patients admitted at the acute, subacute, and chronic stages are presented in Table 1. The average age at the time of injury was ~35 years; this was similar in all three groups. Patients were predominantly male. More patients with TBI (60–70%) were admitted in the acute and subacute stages, while more patients with non-TBI (67%) were admitted in the chronic stage. The program accepted patients from diverse ethnic groups. The proportion of MCS was higher than the proportion of VS in the acute and subacute stages, and lower in the chronic stage, based on CRS-R on admission. CMI was on average ~2.4, which is significantly higher than average CMI in the institution's general brain injury services (1.7–1.8) and the national score (1.3–1.4) in 2014–2018 (Supplementary Figure 1) (20). Most patients (91%) received 1–2 phases of inpatient rehabilitation. Specifically, most patients received 2–3 months' Phase I specialized DoC inpatient rehabilitation (on average 86.4 ± 69.1 days) and a total of 3–4 months' inpatient rehabilitation (on average 105.8 ± 86.2 days) when subsequent admissions were included. Those admitted in the acute stage had the shortest average length of stay for Phase I and for total inpatient rehabilitation. Acute unplanned transfer for emergencies occurred in 30–50% of the patients, with the highest incidence and acuity rates found in patients admitted in the subacute stage.

**Table 1:** Demographics and status of all patients admitted at the acute, subacute, and chronic stages.

	**Full cohort (*N =* 137)**	**Acute (*N =* 20)**	**Subacute (*N =* 84)**	Chronic (*N =* 33)^*^
Age at the time of injury (years)	35.8 ± 15.0	35.8 ± 18.0	35.7 ± 14.4	36.3 ± 15.3
Gender [male (%); female (%)]	103 (75.2%); 34 (24.8%)	17 (85.0%); 3 (15.0%)	60 (71.4%); 24 (28.6%)	26 (78.8%); 7 (21.2%)
Etiology [TBI (%); non-TBI (%)]	81 (59.1%); 56 (40.9%)	12 (60.0%); 8 (40.0%)	58 (69.0%); 26 (31.0%)	11 (33.3%); 22 (66.7%)
**Ethnicity (** ***N*** **, %)**				
White/Caucasian	74 (54.0%)	9 (45.0%)	45 (53.6%)	20 (60.6%)
Hispanic	24 (17.5%)	7 (35.0%)	15 (17.9%)	2 (6.1%)
African-American	22 (16.1%)	3 (15.0%)	14 (16.7%)	5 (15.1%)
Middle Eastern	10 (7.3%)	0	4 (4.8%)	6 (18.2%)
Asian	4 (2.9%)	1 (5.0%)	3 (3.6%)	0
Pacific Islander	2 (1.5%)	0	2 (2.4%)	0
Mixed	1 (0.7%)	0	1 (1.2%)	0
Days since injury on admission	241.4 ± 538.0	19.4 ± 5.9	78.9 ± 65.2	789.5 ± 899.7 Median: 428 (IQR: 211, 1036)
Diagnosis on admission [MCS (%); UWS/VS(%)]	74 (54.0%); 63 (46.0%)	14 (70.0%); 6 (30.0%)	46 (54.8%); 38 (45.2%)	14 (42.4%); 19 (57.6%)
Case Mix Index (CMI)	2.4 ± 0.3 Min 1.6 Max 3.1	2.5 ± 0.3 Min 1.6 Max 3.1	2.4 ± 0.3 Min 1.8 Max 3.1	2.3 ± 0.3 Min 1.7 Max 2.8
**Admission phases (** ***N*** **)**				
Phase I	92	14	52	26
Phase II	32	5	22	5
Phase III	8	0	7	1
Phase IV	3	1	1	1
Phase V	1	0	1	0
Phase VI	1	0	1	0
Total inpatient rehab. days	105.8 ± 86.2	75.8 ± 35.3	117.3 ± 87.9	94.6 ± 99.0 Median: 67 (IQR: 38, 112)
Phase I inpatient days	86.4 ± 69.1	65.3 ± 29.6	91.7 ± 64.6	85.8 ± 92.8 Median 58 (IQR 33, 92)
Acute unplanned transfer (N, %)	57 (41.6%)	7 (35.0%)	38 (45.2%)	12 (36.4%)
Among unplanned transfers, required ICU level of care (N, %)	25 (18.2%)	1 (14.3%)	19 (50.0%)	5 (41.7%)

^*^Including 20 patients who were admitted 1 year after their initial brain injuries.

### Functional recovery rates of patients with DoC admitted at different stages post-injury

**Figure 1:**
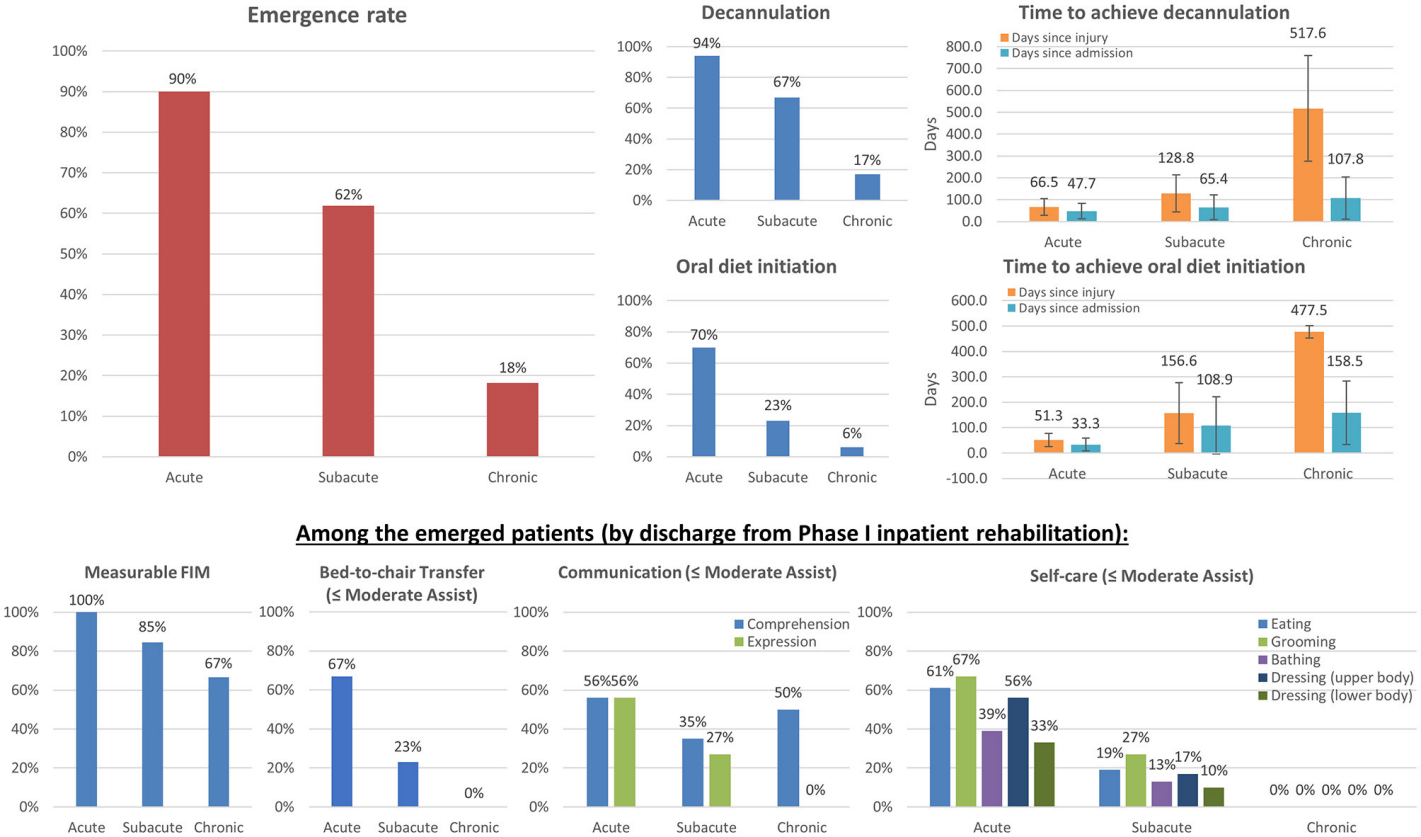
Functional recovery among patients with DoC admitted at different stages post-injury. The total numbers of patients included at the acute, subacute, and chronic stages were 20, 84, and 33, respectively. The left upper panel shows the overall emergence rates (N=18, 52, and 6, respectively). The four right upper panels show the overall decannulation and oral diet initiation rates and corresponding time to those achievements after injury and after admission. The four bottom panels show the percentages of patients undergoing meaningful improvements in transfer, communication, and self-care among emerged patients, requiring only moderate assistance or less (by the end of Phase I inpatient rehabilitation). The results demonstrate that specialized intensive inpatient rehabilitation is crucial for functional recovery from DoC with measurable and meaningful gains.

Functional recovery rates of patients with DoC admitted at different stages post-injury are presented in Figure 1 and Table 2. Almost all patients admitted at the acute stage achieved eMCS, as did over half of patients admitted at the subacute stage. Most patients exhibited measurable improvement on FIM items by the time of discharge from Phase I inpatient rehabilitation. Additionally, 18% of patients admitted at the chronic stage achieved emergence, and two-thirds of this group exhibited measurable improvement on FIM items. Among patients who achieved emergence, by the end of Phase I inpatient rehabilitation, ~60% of patients admitted at the acute stage and 20% of patients admitted at the subacute stage required moderate assistance or less in bed-to-chair transfer, communication, eating, grooming, and upper body dressing. Patients admitted at the chronic stage were very motorically impaired; however, 50% of this group were able to comprehend with moderate assistance or less.

**Table 2:** Functional recovery among patients with DoC admitted at different stages post-injury.

	**Acute (*N =* 20)**	**Subacute (*N =* 84)**	**Chronic (*N =* 33)**
**Emergence rate**	**90.0% (18/20** ^**a**^**)**	**61.9% (52/84)**	**18.2% (6/33)**
**Among emerged patients (by the time of discharge from**			
**Phase I inpatient rehabilitation):**			
Measurable FIM^*^	100.0% (18/18)	84.6% (44/52)	66.7% (4/6)
FIM (subtotal)	33.2 ± 15.2	20.8 ± 11.1	14.7 ± 2.7
FIM (bed/chair transfer)	3.1 ± 1.8	1.7 ± 1.2	1.0 ± 0.0
FIM (locomotion)	3.1 ± 2.1	1.8 ± 1.5	1.0 ± 0.0
FIM (communication)	6.1 ± 3.4	4.7 ± 2.4	3.7 ± 1.5
FIM (social cognition)	7.5 ± 4.1	4.9 ± 2.6	4.0 ± 1.3
FIM (self-care)	13.5 ± 7.2	7.9 ± 5.3	5.0 ± 2.0
**Required moderate assistance or less (FIM score** ≥**3) by the end of**			
**Phase I inpatient rehabilitation):**			
Bed-to-chair transfer	66.7% (12/18)	23.1% (12/52)	0% (0/6)
Locomotion^**^	55.6% (10/18)	17.3% (9/52)	0% (0/6)
Comprehension	55.6% (10/18)	34.6% (18/52)	50% (3/6)
Expression	55.6% (10/18)	26.9% (14/52)	0% (0/6)
Eating	61.1% (11/18)	19.2% (10/52)	0% (0/6)
Grooming	66.7% (12/18)	26.9% (14/52)	0% (0/6)
Bathing	38.9% (7/18)	13.5% (7/52)	0% (0/6)
Dressing (upper body)	55.6% (10/18)	17.3% (9/52)	0% (0/6)
Dressing (lower body)	33.3% (6/18)	9.6% (5/52)	0% (0/6)
**Decannulation rate**	**94.1% (15/16** ^**b**^**)**	**66.7% (48/72** ^**c**^**)**	**17.2% (5/29** ^**d**^**)**
Potential rate^***^	100% (16/16)	73.6% (53/72)	31.0% (9/29)
Days since injury (min, max)	66.5 ± 38.5 (30, 179)	128.8 ± 85.0 (39, 515)	517.6 ± 241.5 (199, 871)
Days since admission (min, max)	47.7 ± 35.3 (17, 153)	65.4 ± 57.0 (3, 314)	107.8 ± 96.1 (30, 267)
**Oral diet initiation rate**	**70.0% (14/20)**	**22.6% (19/84)**	**6.1% (2/33)**
Achieved adult regular diet	45.0% (9/20)	9.5% (8/84)	3.0% (1/33)
Achieved dysphagia diet	25.0% (5/20)	13.1% (11/84)	3.0% (1/33)
Days since injury(min, max)	51.3 ± 25.6 (29, 124)	156.6 ± 120.4 (43, 549)	460.0 and 495.0
Days since admission(min, max)	33.3 ± 24.9 (11, 107)	108.9 ± 112.4 (5, 487)	247.0 and 70.0

^a^One of the non-emerged patients emerged after discharge per note, which brings the rate up to 95.0%. ^b^Three patients were extubated before admission; one patient was not intubated. ^c^Twelve patients were extubated before admission. ^d^Four patients were extubated before admission. ^*^Measurable FIM indicates that a patient scored above 1 on any one of the FIM items. FIM subtotal score range: 12–84; self-care score range: 5–35; bed/chair transfer score range: 1–7; locomotion score range: 1–7; communication score range: 2–14; social cognition score range: 3–21. ^**^Walking or at wheelchair level, whichever scores better. ^***^Including those undergoing capping trials and tolerating a speaking valve by the time of discharge. The bold values were intended to show/distinguish the hierarchy.

Almost all patients admitted at the acute stage were decannulated. This was achieved on average 1.5 months after admission and ~2 months after the initial injury. Approximately 67% of patients admitted at the subacute stage were decannulated. This was achieved on average 2 months after admission and ~4 months after the initial injury. Finally, ~17% of patients admitted at the chronic stage achieved decannulation, while an additional 14% had the potential to be decannulated (when including those undergoing capping trials and tolerating a speaking valve by the time of discharge). This was achieved on average 3.5 months after admission and nearly 1.4 years after the initial injury. There were wide variations among individual cases in the time needed to achieve decannulation. A weak positive correlation was found between days since injury on admission and days to achieve decannulation after admission (*r*_*s*_ = 0.308, *p* = 0.009).

Approximately 70% of patients admitted at the acute stage achieved initiation of an oral diet. This was achieved on average 1 month after admission and ~2 months after the initial injury. Nearly two-thirds of this group achieved a regular diet by the time of final discharge. Approximately 23% of patients admitted at the subacute stage achieved initiation of an oral diet. This was achieved on average 3 months after admission and ~5 months after the initial injury. Nearly half of this group achieved a regular diet by the time of final discharge. Only two patients (6%) who were admitted at the chronic stage achieved initiation of an oral diet; they did so over 2 months and 8 months after admission, which was nearly 1.3 years after their initial brain injuries. A strong positive correlation was found between days since injury on admission and days to achieve initiation of an oral diet after admission (*r*_*s*_ = 0.606, *p* < 0.001).

### Functional achievements of patients with DoC related to TBI and non-TBI

**Figure 2:**
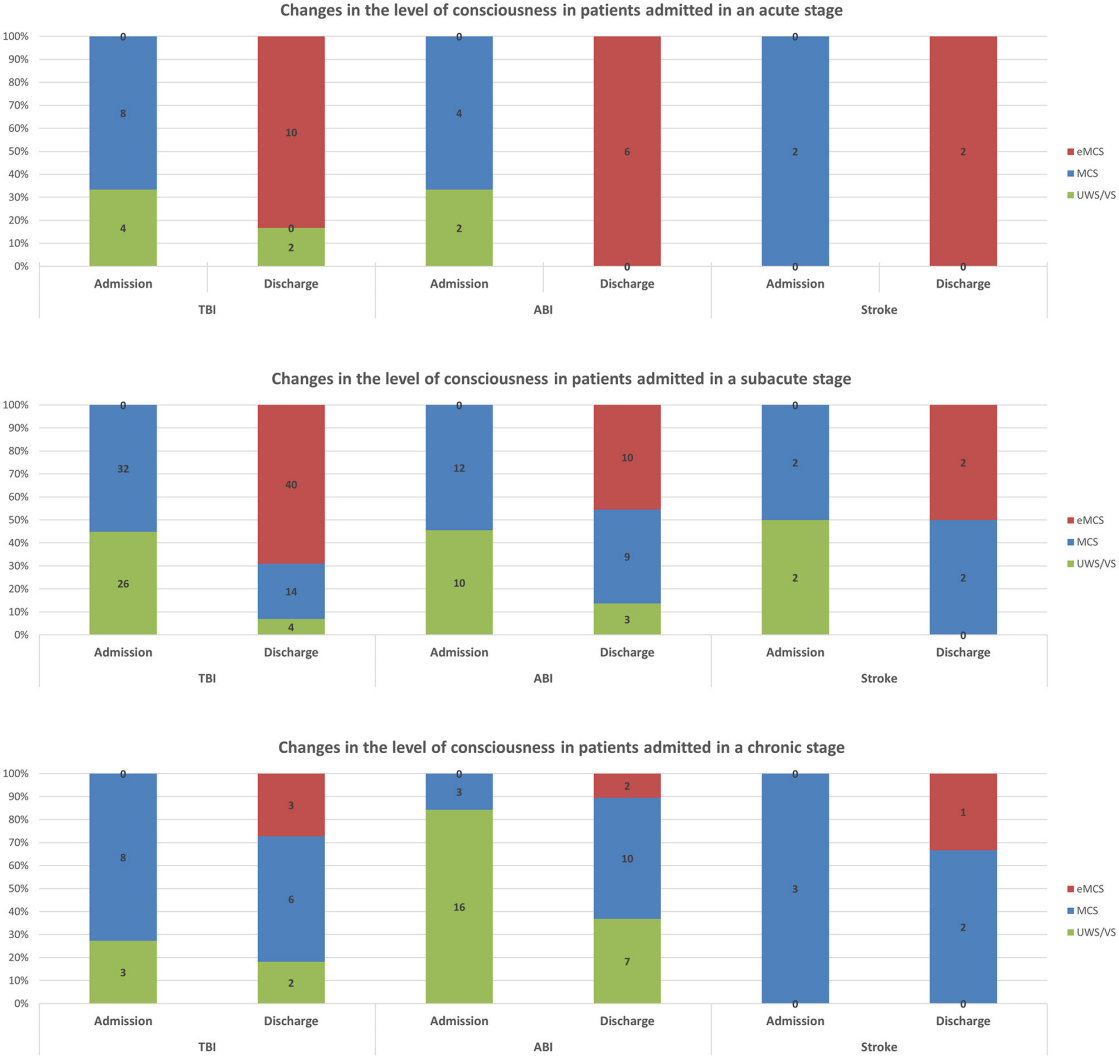
Changes in level of consciousness following specialized acute inpatient rehabilitation among patients with different etiologies and admitted at different stages post-injury.

Changes in the level of consciousness following specialized acute inpatient rehabilitation among patients with different etiologies and admitted at different stages post-injury are presented in Figure 2. As mentioned earlier, among all etiology groups, almost all patients admitted at the acute stage underwent emergence. The emergence rate decreased significantly with increasing chronicity among all etiology groups. For TBI, the rate decreased from 83.3% (10/12) among those admitted at the acute stage to 69.0% (40/58) among those admitted at the subacute stage, and to 27.3% (3/11) among those admitted at the chronic stage. For hypoxic brain injury (also referred to as anoxic brain injury, ABI), the rate decreased from 100.0% (6/6) among those admitted at the acute stage to 45.5% (10/22) among those admitted at the subacute stage, and to 10.5% (2/19) among those admitted at the chronic stage. For stroke, the rate decreased from 100.0% (2/2) among those admitted at the acute stage to 50.0% (2/4) among those admitted at the subacute stage, and to 33.3% (1/3) among those admitted at the chronic stage. Another significant proportion of patients admitted at the chronic stage improved from UWS/VS to MCS, especially in the ABI group.

**Figure 3:**
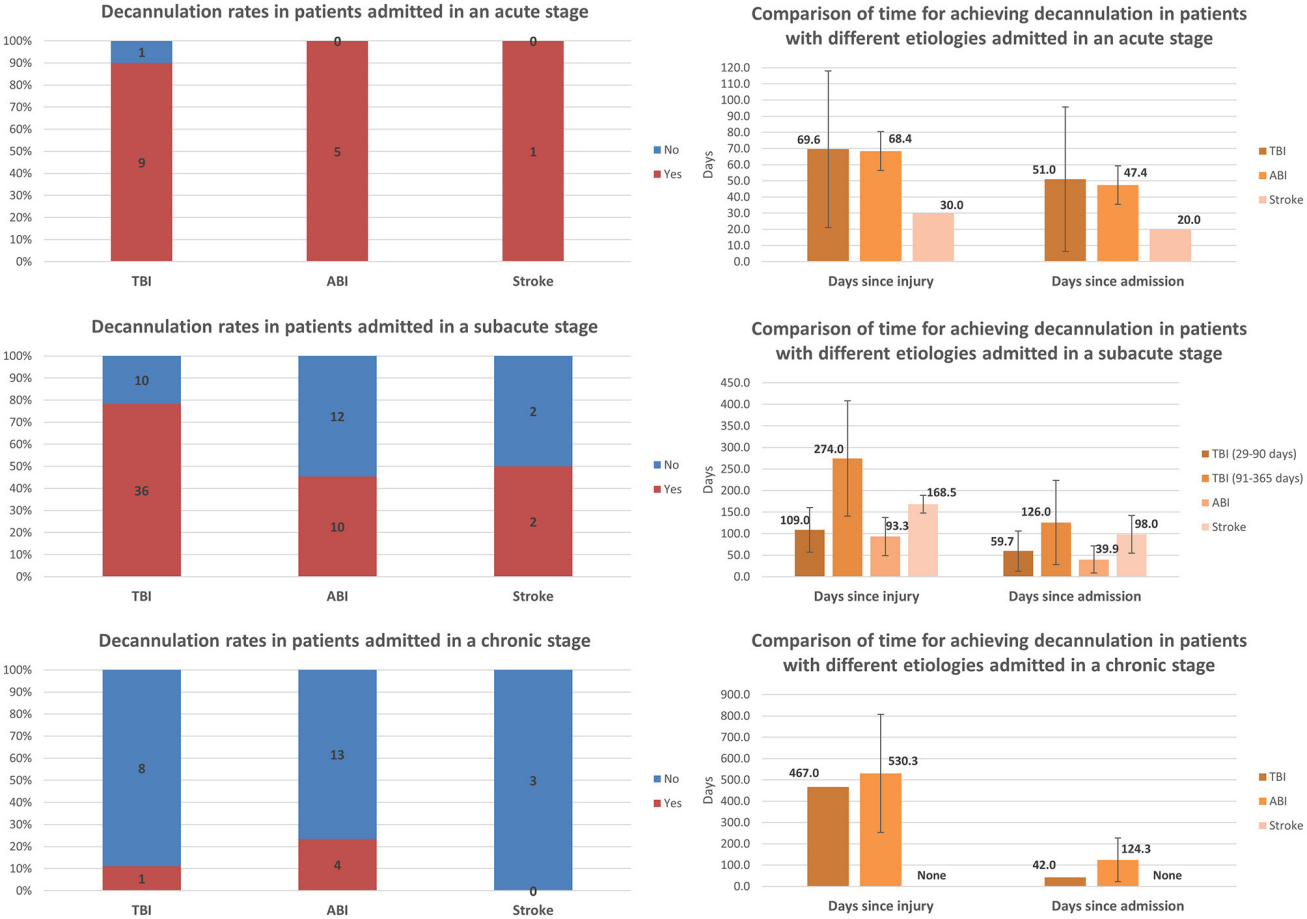
Comparison of decannulation rates and time to achieve decannulation among patients with different etiologies and admitted at different stages post-injury (error bars represent standard deviations).

Comparisons of decannulation rates and time to achieve decannulation among patients with different etiologies and admitted at different stages post-injury are presented in Figure 3. Among all etiology groups, almost all patients admitted at the acute stage achieved decannulation. Decannulation rates decreased significantly with increasing chronicity among all etiology groups. For TBI, the rate decreased from 90.0% (9/10) among those admitted at the acute stage to 78.3% (36/46) among those admitted at the subacute stage, and to 11.1% (1/9) among those admitted at the chronic stage. For ABI, the rate decreased from 100.0% (5/5) among those admitted at the acute stage to 45.5% (10/22) among those admitted at the subacute stage, and to 23.5% (4/17) among those admitted at the chronic stage. For stroke, the rate decreased from 100.0% (1/1) among those admitted at the acute stage to 50.0% (2/4) among those admitted at the subacute stage, and to zero (0/3) among those admitted at the chronic stage. The time to achieve decannulation was similar for TBI and ABI patients who were admitted at the acute stage, on average ~1.5 months after admission and 2 months after injury. The same pattern was found among patients admitted at the subacute stage when comparing only TBI and ABI patients with the same post-injury period of 29–90 days: the achievement was made on average ~1.5–2 months after admission and 3 months after injury. Only one TBI patient and two ABI patients admitted at the chronic stage achieved decannulation, at significantly different periods since admission but at a similar amount of time (approximately 1.4 years) post-injury.

**Figure 4:**
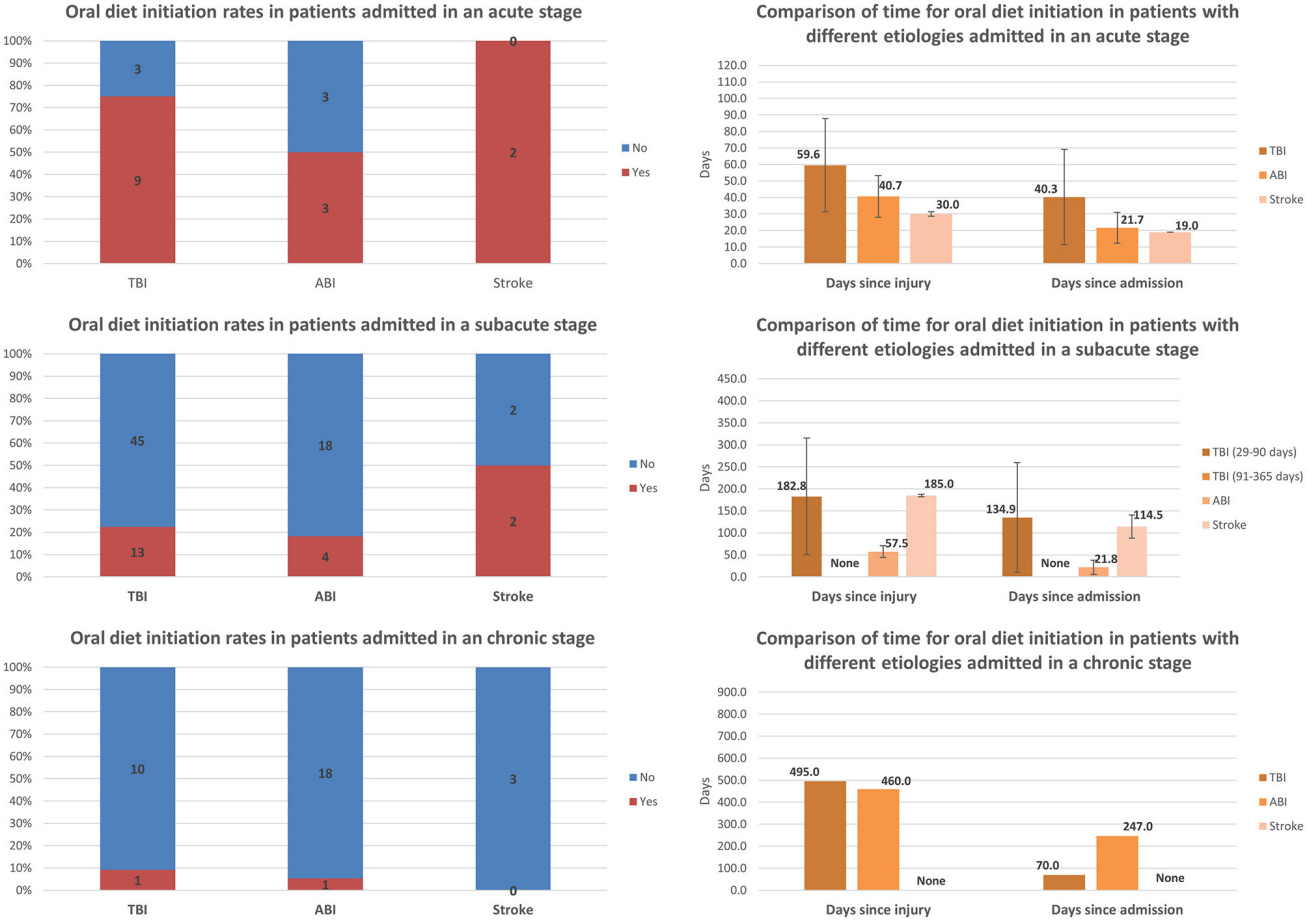
Comparison of rates of oral diet initiation and the time to achieve oral diet initiation among patients with different etiologies and admitted at different stages post-injury (error bars represent standard deviations).

Comparisons of oral diet initiation and the time to achieve oral diet initiation among patients with different etiologies and admitted at different stages post-injury are presented in Figure 4. Most of the TBI patients and half of the ABI patients admitted at the acute stage achieved initiation of an oral diet. The oral diet initiation rate decreased significantly with increasing chronicity among all etiology groups. For TBI, the rate decreased from 75.0% (9/12) among those admitted at the acute stage to 22.4% (13/58) among those admitted at the subacute stage, and to 9.1% (1/11) among those admitted at the chronic stage. For ABI, the rate decreased from 50.0% (3/6) among those admitted at the acute stage to 18.2% (4/22) among those admitted at the subacute stage, and to 5.3% (1/19) among those admitted at the chronic stage. For stroke, the rate decreased from 100.0% (2/2) among those admitted at the acute stage to 50.0% (2/4) among those admitted at the subacute stage, and to zero (0/3) among those admitted at the chronic stage. Interestingly, the time to achieve oral diet initiation appeared to be shorter in ABI than in TBI patients among those admitted at the acute and subacute stages. Only one TBI patient and one ABI patient admitted at the chronic stage achieved an oral diet; these patients did so after significantly different periods following admission but after a similar amount of time (approximately 1.3 years) post-injury.

## Discussion

The results objectively demonstrate functional recovery among persons with DoC following active management and intensive therapies in an acute inpatient rehabilitation program. Almost all patients admitted at the acute stage achieved eMCS (90%) and decannulation (94%); 70% achieved an oral diet; and, ~60% only required moderate assistance or less in bed-to-chair transfer, communication, and self-care using the upper limbs by the end of Phase I inpatient rehabilitation. Rates of functional achievement decreased, and more time was required for these achievements, with increasing chronicity. This was observed in all functional domains and in each etiology group. In this program, patients with TBI and ABI had comparable recovery rates when admitted at the acute or subacute stage. It is also worth noting that a small proportion of persons with chronic DoC made a meaningful functional recovery. The results demonstrate the effectiveness and efficiency of a specialized inpatient DoC rehabilitation program. In concert with the 2018 AAN/ACRM/NIDILRR practice guidelines for DoC, the results support the utility of inpatient rehabilitation for persons with DoC, as evidenced by their functional improvements measured by and beyond the FIM. The results justify the claim that persons with DoC meet the medical necessity requirements for inpatient rehabilitation services regulated by the Centers for Medicare and Medicaid Services (12), specifically regarding “*active*” participation in a sufficient amount of therapy and undergoing significant “*measurable improvements”* as a result of the intensive rehabilitation program.

Furthermore, initiation of oral diet and/or decannulation is indicative not only of improved swallowing and respiratory status, but also of improved voluntary secretion management, airway protection, and reduced risk of aspiration and subsequent pulmonary complications; these improvements carry implications for prognosis as well as healthcare costs. Requiring moderate assistance or less in functional tasks is significantly meaningful to caregivers and could be viewed as a meaningful reduction in care burden. The functional items on which data were collected in the study represent different goals and outcome measures that need to be developed to appraise the rehabilitative benefits of this type of program for patients with DoC. The corresponding clinical results could be used as benchmarks for updated appraisal mechanisms. The study adds practical value and actionable suggestions to the proposed minimum competency recommendations for DoC rehabilitation programs (16).

For recovery, time is a critical factor. Patients admitted at the acute stage had a higher likelihood of achieving emergence, decannulation, and oral diet initiation than those admitted at the subacute and chronic stages. This was the case among patients with DoC related to TBI, ABI, and stroke. Our results showed that it took longer to achieve decannulation and initiation of an oral diet as chronicity increased. The durations of Phase I inpatient rehabilitation stay and total inpatient rehabilitation stay were also noted to be significantly shorter in patients admitted at the acute stage. Therefore, as suggested earlier (11, 15), specialized intensive inpatient rehabilitation is as crucial and time-sensitive for functional recovery in these cases as it has been indicated to be in cases of other types of less severe brain injuries. It is important to emphasize that persons with chronic DoC should not be overlooked under the current healthcare system, as some may be misdiagnosed or suboptimally treated in the acute or subacute stages (21). They may possess the potential to make meaningful improvements under appropriate care (22, 23). Our results revealed a small subset of patients who were found to be fully conscious and made significant functional gains beyond the standard measures used for regulation. In addition, our results present a possible clinical scenario of disproportionate recovery between the mind and the body in the chronic stage, raising concerns about negligence in clinical care and covert suffering.

Providing intensive rehabilitation services is equally important for DoC caused by any etiology. In this study, the time to achieve decannulation was similar in cases of TBI and ABI for patients admitted at both the acute and the subacute stages (in the latter case, when pairing on number of days post-injury). Interestingly, the time to achieve oral diet initiation was shorter for ABI patients than for TBI patients at both the acute and the subacute stages (again, in the latter case, when pairing on number of days post-injury). This finding is distinct from the existing impression of the prognosis of ABI-related DoC.

Our results also support the view that the outcomes of persons with a DoC are not universally poor, and prognostic information should be given cautiously within the first 28 days post-injury (15). Acute inpatient rehabilitation should be provided to patients who still have a DoC following acute care but have achieved medical stability. A delay in providing, or the absence of, this type of care is likely to reduce the chance of functional recovery or prolong the recovery process. It is unclear whether it is the initial severity of the brain injury itself or the delay in rehabilitative interventions that leads to an arrest in recovery in the chronic stage. Delay in care will also increase the risk of medical and musculoskeletal complications, increase the financial burden, and potentially bring with it other ethical and legal challenges. In our previous preliminary study, financial barriers (including insurance denial, a lack of covered benefits, and out-of-network care) accounted for over 40% of denials of referral to an acute inpatient DoC rehabilitation program (13). There is an urgent need to update acute inpatient rehabilitation admission criteria and outcome measures to provide appropriate rehabilitative care to these patients and to avoid undue complications resulting from misdiagnosis and negligence of care. More studies from a clinical rehabilitation perspective are needed.

### Limitations

Even though the data adopted in the study were objective in nature, several limitations must be mentioned. First, without a control group, spontaneous recovery could be a confounding factor, especially in the acute and subacute stages. However, improvements observed in the chronic stage supported the effectiveness of inpatient rehabilitation management. Second, it is possible that the patients were unable to be admitted earlier due to the severity and acuity of their medical conditions. In our preliminary study, nearly 25% of referrals were deferred due to medical instability (for example, a patient was medically stable when the initial referral was placed, but their condition subsequently changed within days while the referral was being processed) (13). Therefore, patients who were admitted at the acute stage may have less severe medical conditions compared to those admitted in a subacute stage, thus resulting in better functional outcomes. This may be indirectly reflected in the acute unplanned transfer data in Table 1. Third, as this study was limited to the information obtained *via* chart review, it is unclear whether patients, especially those admitted at the chronic stage, received rehabilitation services at other facilities. The quality and quantity of rehabilitation services accessed at other facilities were also unmeasurable. This may affect the validity of the conclusions drawn in the study. Fourth, the scope of our study may be skewed by the fact that only a very small percentage of patients with DoC are referred and accepted to receive acute inpatient rehabilitation services—the “tip of the iceberg”—as the majority of these patients are more likely to be discharged to long-term care facilities under “custodial care,” without any rehabilitative interventions. Therefore, our scope may be subject to survivorship bias. Beyond these issues, the sample size became smaller after stratification, which means that the findings warrant further investigation with a larger sample size or a systemic national registry.

## Conclusion

Specialized intensive inpatient rehabilitation is crucial and time-sensitive for functional recovery from DoC. Providing such a level of rehabilitative care is equally important for DoC caused by TBI and by hypoxic–ischemic brain injury. Specific goals and different outcome measures (e.g., consciousness level, decannulation, and oral diet initiation) need to be developed to appraise the benefits of acute inpatient rehabilitation for DoC.

## Author's note

The results of this study were partially submitted as an abstract to AAP 2023 and IBIA 2023.

## Data availability statement

The raw data supporting the conclusions of this article will be made available by the authors, without undue reservation.

## Ethics statement

The studies involving human participants were reviewed and approved by the University of Texas Health Science Center at Houston Committee for the Protection of Human Subjects (HSC-MS-18-0198). Written informed consent for participation was not required for this study in accordance with the national legislation and the institutional requirements.

## Author contributions

Conceptualization, project administration, and methodology: BZ, KO'B, and SL. Data acquisition: BZ, BC, and CR. Formal analysis: BZ and BC. Writing–original draft preparation: BZ, KO'B, and JW. Writing–review and editing: BZ, KO'B, JW, BC, CR, SL, and SK. All authors contributed to the article and approved the submitted version.

## Supplementary material

Supplementary Figure 1 Case Mix Index for the institution's general brain injury services (blue lines; average = 1.7–1.8) and national brain injury services (green lines; average = 1.3–1.4) in 2014–2018.
