# Supplementary figures and images for: Specialized intensive inpatient rehabilitation is crucial and time-sensitive for functional recovery from disorders of consciousness
Source: Front Neurol. 2023 Apr 6;14:1126532. doi: 10.3389/fneur.2023.1126532 (PMC10117854; doi:10.3389/fneur.2023.1126532)

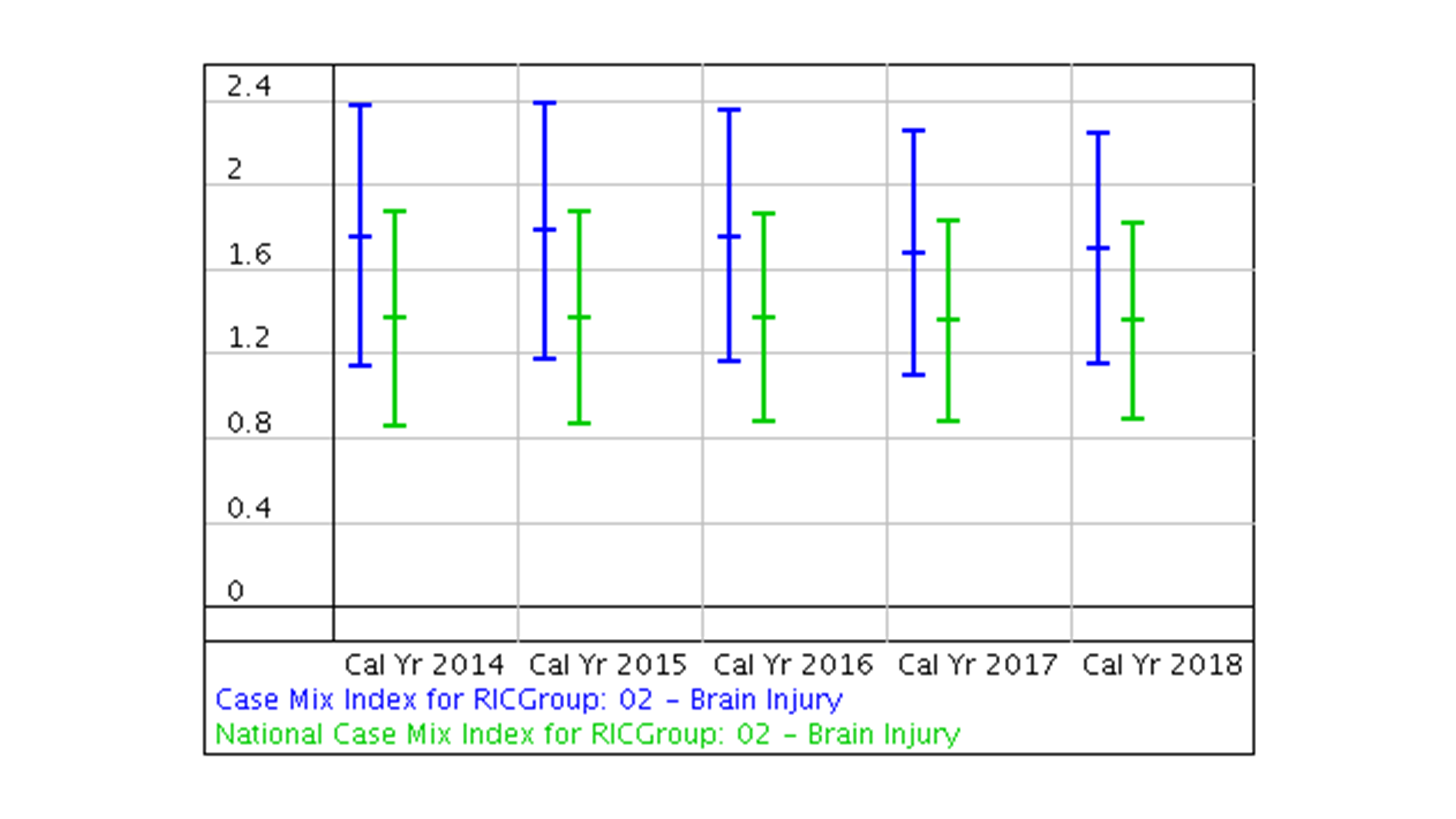

Supplement: Supplementary Figure 1 — Case Mix Index for the institution's general brain injury services (blue lines; average = 1.7–1.8) and national brain injury services (green lines; average = 1.3–1.4) in 2014–2018. [file Image_1.TIF]
